# Use of the precision-fed cecectomized rooster assay to determine standardized amino acid digestibility, true metabolizable energy content, and digestible indispensable amino acid scores of plant-based protein by-products used in canine and feline diets

**DOI:** 10.1093/tas/txab025

**Published:** 2021-02-16

**Authors:** Lauren M Reilly, Patrick C von Schaumburg, Jolene M Hoke, Gary M Davenport, Pamela L Utterback, Carl M Parsons, Maria R C de Godoy

**Affiliations:** 1 Department of Animal Sciences, University of Illinois, Urbana, IL, 61801, USA; 2 ADM, Decatur, IL 62526, USA

**Keywords:** by-product, cat, cecectomized rooster, dog, plant, protein quality

## Abstract

Traditionally, protein by-products from oil seeds and cereal grains have been used in pet foods as sustainable, inexpensive, and protein-rich ingredients. However, the on-going demonization of soy- and corn-based ingredients continue to hinder their use in pet food and treat formulations. Ideally, the further demonstration of their protein quality and nutrient composition may encourage their favorable return as acceptable ingredients in pet foods and treats. Therefore, the objectives of this study were to determine the macronutrient composition, indispensable amino acid profile, standardized amino acid digestibility, true metabolizable energy content corrected for nitrogen (TMEn), and digestible indispensable amino acid scores (DIAAS-like) of soy flakes (SF), peanut flour (PF), soybean meal (SBM), and corn gluten meal (CGM). Standardized amino acid digestibility was assessed using the precision-fed cecectomized rooster assay. All test ingredients demonstrated a profile of highly digestible indispensable amino acids except for lysine in PF, which was lowest (*P* < 0.05) at 45.5%. The SBM and CGM had the highest (*P* > 0.05) digestibilities of indispensable amino acids. A DIAAS-like value was calculated for each ingredient using either AAFCO (2020) recommended values or NRC (2006) recommended allowances as the reference protein pattern. For adult dogs compared to AAFCO recommended values, the first-limiting amino acid was lysine for PF and CGM but it was methionine for SF and SBM. For adult cats compared to AAFCO recommended values, the first-limiting amino acid was lysine for PF and CGM but it was threonine for SF. There was no first-limiting amino acid in SBM for cats as DIAAS-like values were over 100% for all indispensable amino acids. The TMEn values were highest (*P* < 0.05) for PF and CGM (4.58 and 4.31 kcal/g [dry matter basis], respectively). The protein quality of these plant-based protein by-products reflects their value as nutritional ingredients for canine and feline diets. However, the prior processing of these by-products must be considered before exposing them to additional processing methods, such as extrusion. Additionally, the inclusion of complementary proteins or supplemental amino acids will be needed to meet all indispensable amino acid requirements for a portion of nutritionally complete and balanced pet food.

## INTRODUCTION

Plant-based protein by-products, also referred to as coproducts, are secondary products formed after the initial processing of the plant for human foods (AAFCO, 2020). The use of these by-products in the pet food industry reduces competition for human-grade protein sources that more pet owners want in their pet foods (Meeker and Meisinger, 2015). This humanization of pet food is a reflection of the increasing anthropomorphism of dogs and cats, creating a demand for easily recognizable ingredients on pet food labels (Carter et al., 2014). Plant-based protein by-products often are identified as the specific fraction of the plant, rather than the generalized term “by-product” in the ingredient name on petfood labels (AAFCO, 2020), helping to improve consumer acceptability of these ingredients in canine and feline diets.

Plant-based protein by-products are produced using a variety of processing methods including milling, flaking, and dehulling that can influence the amino acid profile and macronutrient composition (Lusas and Riaz, 1995; Moniruzzaman et al., 2020). While these plant-derived by-products are readily available and protein-rich ingredients, recent trends in the pet food industry have limited their inclusion in canine and feline diets. For many pet owners, the absence of by-products in a pet food equates to a higher-quality product. The petfood industry would benefit from additional research characterizing the nutritional quality of these plant-based ingredients to stimulate their favorable return back into the pet food industry.

The objectives of this study were to characterize select plant-based protein by-products based on macronutrient composition, standardized amino acid digestibility, and digestible indispensable amino acid scores (DIAAS-like) of soy flakes (SF), soybean meal (SBM), corn gluten meal (CGM), and peanut flour (PF). It was hypothesized that these plant-based protein by-products would be variable in their macronutrient and amino acid composition but each would be a highly concentrated and digestible source of protein for use in pet foods and treats.

## MATERIALS AND METHODS

The animal procedures and protocols used in this study were approved by the Institutional Animal Care and Use Committee at the University of Illinois at Urbana-Champaign. All methods were performed in accordance with the United States Public Health Service Policy on Humane Care and Use of Laboratory Animals.

### Sample Preparation and Chemical Analysis

All test ingredients (SF, PF, SBM, and CGM) were supplied by ADM (Decatur, IL). Each sample was ground through a 2-mm screen in a Wiley mill (model 4; Thomas Scientific, Swedesboro, NJ) and then analyzed in duplicate for chemical composition. Dry matter (DM), ash, and organic matter (OM) were analyzed according to AOAC (2006; methods 934.01 and 942.05). Crude protein (CP) was calculated from Leco (TruMac N, Leco Corporation, St. Joseph, MI) with total nitrogen being determined according to AOAC (2006; method 992.15). Gross energy (GE) was measured using bomb calorimetry (Model 6200, Parr Instruments Co., Moline, IL). Acid hydrolyzed fat (AHF) measured total fat content according to AACC (1983) and Budde (1952). Total dietary fiber (TDF) was analyzed according to Prosky et al. (1992). Complete amino acid profiles were measured according to AOAC (2007).

### Precision-fed Rooster Assay

A precision-fed rooster assay was conducted using 16 cecectomized, single-comb White Leghorn roosters (four roosters/treatment) according to Parsons et al. (1985). A temperature-controlled room on a 16 h light and 8 h dark schedule was used to house the roosters in wire-floored cages. After being fasted for 26 h, roosters were crop intubated with 30 g of a 1:1 mixture of the test ingredient and ground corn. Excreta were quantitatively collected for 48 h, freeze-dried as a single composite sample, and ground to uniform particle size. Excreta were analyzed for amino acids (AOAC, 2007). Standardized amino acid digestibility was calculated using endogenous values that were derived across multiple roosters over several years according to Sibbald (1979):

(Eq. 1) Step 1:


$$\begin{array}{l} \text{Mixed Amino Acid Digestibility}(\%)\\ =\frac{\mathrm{FAA}-\mathrm{EAA}+\mathrm{EndAA}}{\mathrm{FAA}}\times 100 \end{array}$$

Step 2:


$$\begin{array}{l} Standardized\;\;\;Amino\;\;\;Acid\;\;\;Digestibility\;\;\;\left(\;\%\;\right)\\ =\;\frac{AAD_{c}-(AAD_{c}-AAD_{m})}{FAA\;\;\;Ratio}\times 100 \end{array}$$

First, a mixed amino acid digestibility was calculated for the combination of corn and test ingredient where FAA is the total amino acids fed; EAA is the total amino acids voided in the excreta; and EndAA is the total endogenous amino acids voided in the excreta of fasted roosters. To correct for the added corn, the standardized amino acid for each test ingredient was calculated where AAD_c_ is the amino acid digestibility of the corn; AAD_m_ is the amino acid digestibility of the mixture; and FAA Ratio is the ratio of the amino acid content (%) of the test ingredient relative to the amino acid content (%) of the mixture of test ingredient and ground corn.

Excreta also were analyzed for true metabolizable energy corrected for nitrogen (TMEn) according to Parsons et al. (1982). The TMEn values were calculated using the following equations:

(Eq. 2) Step 1: 


$$\begin{array}{ll} Mixed\;\;\;TMEn\;\;\;\left(kcal/g\right) & \\ =\;\frac{FE_{fed}-\left(EE_{fed}+8.22\left(N_{fed}\right)\right)+\left(EE_{fasted}+8.22\left(N_{fasted}\right)\right)}{FI} \end{array}$$

Step 2: 


$$\begin{array}{l} TMEn\;\;\;\left(kcal/g\right)=\;\;\;TMEn_{c}-\frac{\left(TMEn_{c}-TMEn_{m}\right)}{0.5} \end{array}$$

A mixed TMEn value was initially calculated for the ground corn and test ingredient mixture. In the above equation, FE_fed_ is the gross energy of the feed (kcal); EE_fed_ and EE_fasted_ is the excreta energy (kcal) of the fed and fasted roosters, respectively; 8.22 is the gross energy per gram of nitrogen of uric acid, N_fed_ and N_fasted_ is the amount of nitrogen (g) retained in the fed and fasted birds, respectively; and FI is feed intake. To derive the TMEn value exclusively for the test ingredient, the equation was corrected for corn. In the above equation, TMEn_c_ is the TMEn of the added ground corn, TMEn_m_ is the mixed TMEn, and 0.5 corrects for the 1:1 mixture of corn and test ingredient.

### DIAAS-like Values

An altered version of digestible indispensable amino acid scores (DIAAS-like) was calculated according to Mathai et al. (2017) to determine protein quality. The DIAAS-like values were calculated using the standardized indispensable amino acid digestibility values from the precision-fed rooster assay. Recommended nutrient values from the Association of American Feed Control Officials (AAFCO, 2020) and recommended nutrient allowances from the National Research Council (NRC, 2006) were used to calculate the reference protein patterns (mg/g) by determining each indispensable amino acid (mg) in 1 g of protein based on canine and feline adult maintenance requirements. The indispensable amino acid (mg) present in 1 g of protein from each test ingredient also was calculated. The DIAAS-like values were calculated using the equation:

(Eq. 3) 


$$\begin{array}{ll} DIAAS-like\;\;\;\left(\;\%\;\right) & \\ =\frac{mg\;\;of\;digestible\;\;AA\;\;in\;\;1\;\;g\;dietary\;\;\;protein\;\;\;}{mg\;\;\;of\;same\;\;AA\;in\;\;1\;\;g\;\;of\;\;reference\;\;protein}\;\times \;100 \end{array}$$

### Statistical Analyses

All data were analyzed in SAS (SAS Institute Inc., version 9.4, Cary, NC) using the Mixed Models procedure. The model for the precision-fed rooster assay was performed with a fixed effect of treatment and a random effect of the rooster. Differences among treatments were reported using a Fisher-protected least significant difference test with a Tukey adjustment to control for a type-1 experiment-wise error. Differences among treatments were considered statistically significant using a probability of *P* < 0.05. The standard errors of the mean (SEM) were reported based on the Mixed Models procedure in SAS.

## RESULTS AND DISCUSSION

### Chemical Composition of Plant-based Protein By-products

The plant-based protein by-products had variable macronutrient composition (Table 1). By nature, by-products are variable due to different processing parameters and the ingredient source. Soy flakes are produced by cracking, dehulling, and flaking whole soybeans. The soy flakes then are defatted, toasted, and cooled to form SBM (Johnson and Smith, 2011). Corn gluten meal is produced during the wet-milling of corn which breaks the corn kernel into starch, protein, and dietary fiber fractions (Moniruzzaman et al., 2020). Lastly, PF is produced commercially through pre-press extraction methods and milling to form a flour from raw or roasted peanuts (Ayres et al., 1974). Additionally, plant-based by-products can vary in chemical composition depending on geographical region, growing environment, and storage conditions (Thakur and Hurburgh, 2007).

**Table 1.** Macronutrient composition of select plant-based protein by-products

Item	Treatment			
	Soy flakes	Corn gluten meal	Soybean meal	Peanut flour
Dry matter (%)	93.7	92.0	90.1	98.8
	%, DMB^*a*^			
Organic matter (%)	93.3	94.4	92.5	96.3
Crude protein (%)	59.5	67.5	52.7	48.6
Acid-hydrolyzed fat (%)	3.1	9.1	3.2	26.1
Total dietary fiber (%)	23.8	20.2	30.3	17.7
Gross energy^*b*^ (kcal/g)	4.6	5.7	4.7	6.1

^*a*^ DMB = dry matter basis.

^*b*^ Gross energy measured by bomb calorimetry.

The DM content of SBM was lowest at 90.1% while PF had the highest DM content at 98.8%. The remaining proximate analysis values are expressed on a dry matter basis (DMB). The OM content followed the same pattern as the DM content with SBM having the lowest OM content of 92.5% and PF having an OM content of 96.3%. A high degree of variation was observed in the CP and AHF contents of these by-products. The CP content of SF was 59.5%, CGM was 67.5%, SBM was 52.7%, and PF was 48.6%. A previous study reported the CP content of corn gluten meal to be 65.3% and soybean meal to be 44.8% (Lee et al., 2019). Soy flakes have previously been reported to have 60.6% CP (Romarheim et al., 2007). The CP content of PF is variable according to previous literature. Ayres and Davenport (1977) reported CP content to be 60% (DMB) in flours while Yu et al. (2006) reported 52.7% (DMB) in defatted raw flour and 54.6% (DMB) in defatted roasted peanut flour.

The AHF content of SF was 3.1%, CGM was 9.1%, SBM was 3.2%, and PF was 26.1%. The peanut processing method retains the oil fraction to create a final product with high lipid content. This oil content must be considered when formulating companion animal diets with PF to avoid higher than expected fat levels in the diet and a greater opportunity for fat oxidation and spoilage (Ayres et al., 1974). Peanut flour from raw peanuts (defatted) has been reported to contain 17.0% fat (Yu et al., 2006). In contrast, soy products (i.e., flakes and meal) are defatted during processing, resulting in low-fat by-products (Johnson and Smith, 2011) that can be more easily incorporated into pet foods and reducing the risk of lipid oxidation. The process of soy flaking facilitates oil extraction by thinning the soybean layers to minimize the distance to the soybean surface (FAO/WHO, 1992). Previous studies have reported the crude fat content of soy flakes to be 2.4% (DMB), slightly lower than the value observed in the current study (Romarheim et al., 2007). Soybean meal has also been previously reported to have an AHF content of 3.2% (Menniti et al., 2014). For CGM, Aslaksen et al. (2007) reported a lipid content of 9.26% which is similar to the value measured in the current study. The GE content of SF and SBM were similar at 4.6 and 4.7 kcal/g, respectively. The GE content of CGM was 5.7 and 6.1 kcal/g for PF, reflective of the higher AHF content. Because plant-based protein by-products include varying fractions of the whole plant, the TDF content can be variable. The TDF content of by-products assessed in this study ranged from 17.7% for PF to 30.3% for SBM. Whole soybeans have been previously reported to contain 32.4% TDF (Aslaksen et al., 2007).

The complete amino acid profile (Table 2) reflects the variation observed in the CP content of the test ingredients. The legume-based protein by-products were lower in methionine (average 0.69%) compared to CGM which had a methionine content of 1.75% (DMB). The lysine content of PF was the lowest at 0.94% compared to SBM (3.44%), SF (3.77%), and CGM (1.25%). As legumes, soy-based protein by-products have higher concentrations of lysine and limited sulfur-containing amino acids making them complementary to corn and other cereal grains (Thakur and Hurburgh, 2007). The lysine content of PF in the current study is higher than peanut flour analyzed by Park et al. (2017) which had a lysine content of 0.4% (DMB). The leucine content of CGM in the present study was more than twice (11.0%) the leucine content of SF (4.6%), SBM (4.0%), and PF (3.1%). Corn contains high concentrations of branched-chain amino acids, particularly leucine, which has been shown to have a beneficial role in glucose homeostasis in rats (Bong et al., 2010). Leucine content in corn gluten hydrolysates was reported to be 11.5% (Bong et al., 2010) and 11.8% in corn gluten meal (Lee et al., 2019).

**Table 2.** Amino acid composition of select plant-based protein by-products

%, DMB^*a*^	Treatment			
	Soy flakes	Corn gluten meal	Soybean meal	Peanut flour
***Indispensable amino acid***				
Arginine	4.29	2.34	3.84	4.82
Histidine	1.56	1.43	1.40	1.08
Isoleucine	2.93	2.96	2.57	1.72
Leucine	4.58	11.04	4.04	3.07
Lysine	3.77	1.25	3.44	0.94
Methionine	0.81	1.75	0.75	0.50
Phenylalanine	3.07	4.31	2.64	2.44
Threonine	2.17	2.17	2.03	1.16
Tryptophan	0.80	0.43	0.73	0.56
Valine	3.08	3.29	2.72	2.11
***Dispensable amino acid***				
Alanine	2.52	5.80	2.30	1.86
Aspartic acid	6.72	4.21	6.06	5.39
Cysteine	0.85	1.25	0.79	0.50
Glutamic acid	10.72	14.28	9.76	8.79
Glycine	2.56	1.98	2.34	2.75
Proline	3.15	6.16	2.74	2.11
Serine	2.24	2.68	2.46	1.72
Tyrosine	2.13	3.46	1.84	1.76
***Total amino acids, %***	58.49	71.52	52.73	43.97

^*a*^MB = dry matter basis.

### Precision-fed Rooster Assay

The precision-fed cecectomized rooster model was used to calculate standardized amino acid digestibility values for each plant-based protein by-product (Table 3). The use of the precision-fed cecectomized rooster model has proven to be an accurate model for estimating canine in vivo nutrient digestibility. Standardized amino acid digestibility values calculated using the cecectomized rooster model have been similar to those calculated using ileal-cannulated dogs (Johnson et al., 1998). The suitability of cecectomized roosters as a model for feline nutrition has not been definitively established. For this assay, it is necessary to mix each test ingredient with an equal amount of ground corn to minimize adherence of the test ingredient to the delivery tube to ensure complete deposition into the crop of the rooster. The corn used in this assay represented a single harvest so its contribution of amino acids and endogenous values could be factored out of the equation to provide standardized amino acid digestibility values attributed solely to the test ingredient.

**Table 3.** Standardized amino acid digestibility values of select plant-based protein by-products determined using the precision-fed cecectomized rooster assay^*a*^

%, DMB^*b*^	Treatment				
Indispensable amino acid	Soy flakes	Corn gluten meal	Soybean meal	Peanut flour	SEM^*c*^
Arginine	85.3^b^	94.0^a^	96.0^a^	91.8^a^	1.019
Histidine	79.4^b^	94.0^a^	94.8^a^	82.5^b^	1.461
Isoleucine	76.1^c^	94.0^a^	93.1^a^	84.5^b^	1.132
Leucine	75.1^d^	97.4^a^	93.0^b^	86.8^c^	0.938
Lysine	78.1^b^	81.8^ab^	92.1^a^	45.5^c^	2.808
Methionine	74.0^d^	96.4^a^	91.3^b^	79.7^c^	1.196
Phenylalanine	78.1^c^	95.7^a^	93.7^a^	90.1^b^	0.862
Threonine	71.6^b^	93.3^a^	92.6^a^	75.4^b^	1.912
Tryptophan	84.7^c^	94.2^a^	96.3^a^	90.1^b^	0.807
Valine	73.5^c^	94.2^a^	92.8^a^	85.1^b^	1.349

^a–d^ Means within a row with different superscript letters are different (*P* < 0.05).

^*a*^ *n* = 4 cecectomized roosters per treatment.

^*b*^ DMB = dry matter basis.

^*c*^ SEM = standard error of the mean.

Results showed the standardized lysine digestibility was lower (*P* < 0.05) for PF (45.5%). Similarly, Park et al. (2017) reported low standardized lysine digestibility for peanut flour in broiler chickens (66.1%) and pigs (66.5%). The decrease in PF lysine digestibility may be due to the presence of Maillard reaction products formed during peanut roasting. Maillard reactions are non-enzymatic reactions that occur between free amino groups and reducing sugars, particularly lysine, due to the presence of an ε-amino group in the side chain (Hemmler et al., 2018). The formation of Maillard reaction products reduces lysine digestibility and limits its availability to the host (van Rooijen et al., 2013). Therefore, the lower lysine digestibility in this study is likely attributed to using a dark-roasted PF. While Maillard reactions occurring during the roasting process are responsible for color and flavor development of PF, it can also negatively impact the protein quality of the ingredient. One study showed that as roast color darkened, the amount of available lysine present in PF decreased (McDaniel et al., 2012). In contrast to SBM which had standardized amino acid digestibility values over 90% for all indispensable amino acids, SF had the lowest (*P* < 0.05) values for 9 of the 10 indispensable amino acids ranging from 71.6% to 85.3% (DMB). Soybeans typically contain high concentrations of anti-nutritional factors, particularly trypsin inhibitors (Gu et al., 2010). These anti-nutritional factors may have influenced the observed standardized amino acid digestibility values of the SF. Antinutritional factors can be reduced or eliminated with heat treatment (Gu et al., 2010), thus the same response was not observed with SBM likely due to its additional processing.

**Figure 1.**
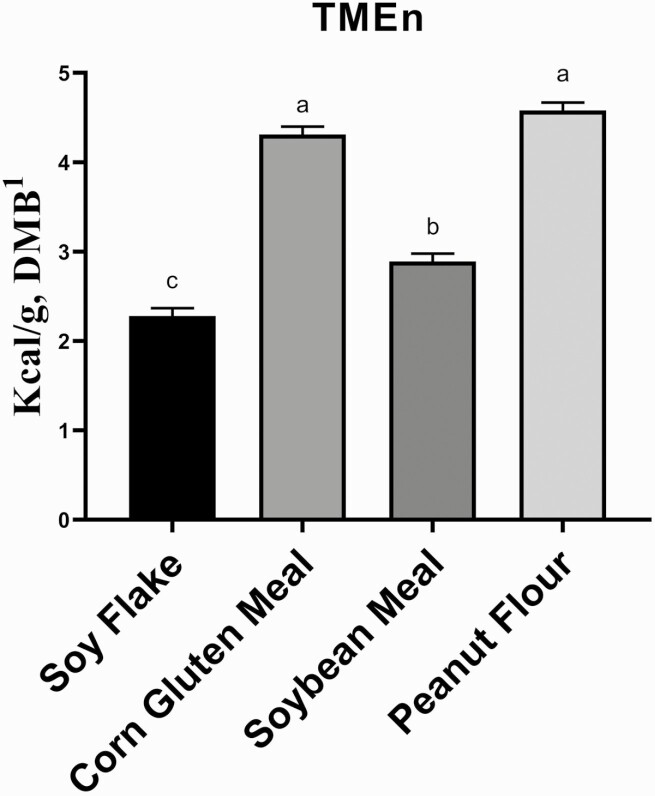
True metabolizable energy content corrected for nitrogen (TMEn) of select plant-based protein by-products. ^a–c^ Means with different superscript letters are different (*P* < 0.05). ^1^ DMB = dry matter basis.

The TMEn values were calculated for each plant-based protein by-product (**Figure 1**). The TMEn values account for endogenous energy losses and higher nitrogen losses in fasted birds compared to fed birds. The correction for these losses provides a more accurate measure of TMEn (Parsons et al., 1982). The TMEn contribution of the ground corn was factored out of the equation in this study. For the test ingredients, PF and CGM had the highest (*P* < 0.05) TMEn values (4.58 and 4.31 kcal/g (DMB), respectively). In contrast, SF had the lowest (*P* < 0.05) TMEn value at 2.28 kcal/g (DMB). Coon et al. (1990) measured the TMEn value of traditional and ethanol-extracted (i.e., oligosaccharide-free) soybean meals. The traditional soybean meal had a TMEn value of 2.79 kcal/g (DMB) and the ethanol-extracted soybean meal had a TMEn value of 3.37 kcal/g (Coon et al., 1990). The plant-based protein by-products used in this study have similar TMEn values to dried distiller’s grain with solubles (DDGS), which range from 2.48 to 3.05 kcal/g (Fastinger et al., 2006). Parsons (1985) also reported a TMEn value for DDGS of 3.16 kcal/g.

### DIAAS-like Values

A DIAAS-reference score was calculated for each indispensable amino acid in the test ingredient. The lowest DIAAS-reference score is the assigned DIAAS-like value for the test ingredient which determines its overall protein quality. Additionally, the lowest DIAAS-like value represents the first-limiting amino acid, recognizing there are no limiting amino acids in a test ingredient if DIAAS-reference scores are over 100% for all amino acids. A high-quality protein source has a DIAAS-like value equal to or greater than 100%, while a protein with a score less than 100 but greater than 50% is considered moderate quality. A protein with a score of less than 50% represents a low-quality protein source which would be inadequate as the sole protein source in a dog or cat food (Mathai et al., 2017).

Traditional DIAAS values are calculated using standardized amino acid digestibility values obtained using ileal-cannulated pigs with the estimated average requirements of 2-5-year-old children as the reference protein (Marinangeli and House, 2017). In this study, the DIAAS-like values were calculated using the standardized amino acid digestibility values from the cecectomized rooster assay and the reference proteins based on AAFCO (2020) recommended values and NRC (2006) recommended allowances for adult dogs (**Tables 4 and 5**) and cats (**Tables 6 and 7**) at maintenance. Unlike the FAO-established method of determining protein quality, known as protein digestibility-corrected amino acid scores (PDCAAS), DIAAS scores are not truncated at 100% which avoids under-estimating the quality of high-quality protein sources (Mathai et al., 2017; FAO/WHO, 1992).

**Table 4.** Digestible indispensable amino acid score (DIAAS)-like^*a*^ values for select plant-based protein by-products compared to AAFCO (2020) recommended values for adult dogs at maintenance

% DMB^*b*^		Treatment			
Indispensable amino acid	Soy flakes	Corn gluten meal	Soybean meal	Peanut flour	SEM^*c*^
Arginine	216.1^c^	114.5^d^	246.1^b^	320.5^a^	1.914
Histidine	195.1^b^	186.7^bc^	232.6^a^	172.0^c^	3.794
Isoleucine	177.1^c^	195.3^b^	215.1^a^	141.7^d^	2.347
Leucine	152.9^c^	421.3^a^	188.8^b^	145.2^c^	2.100
Lysine	140.7^b^	43.1^c^	171.2^a^	25.1^d^	2.598
Methionine	54.6^c^	135.5^a^	70.5^b^	44.5^d^	1.028
Phenylalanine	160.3^c^	243.3^a^	187.0^b^	180.0^b^	1.873
Threonine	97.9^c^	112.4^b^	133.8^a^	67.5^d^	2.117
Tryptophan	128.1^b^	67.5^d^	150.1^a^	116.9^c^	0.757
Valine	139.1^b^	167.9^a^	175.2^a^	135.1^b^	2.353

^a–d^ Means within a row with different superscript letters are different (*P* < 0.05)

^*a*^ DIAAS-like (%) = [(mg of digestible indispensable amino acid in 1 g of dietary protein)/mg of same indispensable AA in 1 g of reference protein)] × 100.

^*b*^ DMB = dry matter basis.

^*c*^ SEM = standard error of the mean.

**Table 5.** Digestible indispensable amino acid score (DIAAS)-like^*a*^ values for select plant-based protein by-products compared to NRC (2006) recommended allowances for adult dogs at maintenance

%, DMB^*b*^	Treatment				
Indispensable amino acid	Soy flakes	Corn gluten meal	Soybean meal	Peanut flour	SEM^*c*^
Arginine	175.6^c^	93.0^d^	199.9^b^	260.4^a^	1.556
Histidine	109.5^b^	104.8^bc^	130.6^a^	96.6^c^	2.131
Isoleucine	98.4^c^	108.5^b^	119.5^a^	78.7^d^	1.303
Leucine	84.9^c^	234.1^a^	104.9^b^	80.7^c^	1.166
Lysine	141.2^b^	43.3^c^	171.8^a^	25.2^d^	2.607
Methionine	30.5^c^	75.7^a^	39.4^b^	24.9^d^	0.574
Phenylalanine	89.5^c^	135.8^a^	104.4^b^	100.5^b^	1.045
Threonine	60.7^c^	69.7^b^	82.9^a^	41.9^d^	1.313
Tryptophan	81.3^b^	42.9^d^	95.3^a^	74.2^c^	0.481
Valine	77.6^b^	93.6^a^	97.7^a^	75.4^b^	1.312

^a–d^ Means within a row with different superscript letters are different (*P* < 0.05).

^*a*^ DIAAS-like (%) = [(mg of digestible indispensable amino acid in 1 g of dietary protein)/mg of same indispensable AA in 1 g of reference protein)] × 100.

^*b*^ DMB = dry matter basis.

^*c*^ SEM = standard error of the mean.

**Table 6.** Digestible indispensable amino acid score (DIAAS)-like^*a*^ values for select plant-based protein by-products compared to AAFCO (2020) recommended values for adult cats at maintenance

%, DMB^*b*^	Treatment				
Indispensable amino acid	Soy flakes	Corn gluten meal	Soybean meal	Peanut flour	SEM^*c*^
Arginine	153.7^c^	81.4^d^	174.9^b^	227.9^a^	1.361
Histidine	173.4^b^	165.9^bc^	206.8^a^	152.9^c^	3.373
Isoleucine	186.9^c^	206.1^b^	227.0^a^	149.6^d^	2.477
Leucine	121.1^c^	333.8^a^	149.6^b^	115.1^c^	1.665
Lysine	154.4^b^	47.3^c^	187.9^a^	27.5^d^	2.850
Methionine	131.0^c^	325.0^a^	168.9^b^	106.7^d^	2.465
Phenylalanine	249.3^c^	378.2^a^	290.8^b^	279.9^b^	2.913
Threonine	92.8^c^	106.5^b^	126.7^a^	63.9^d^	2.005
Tryptophan	185.2^b^	97.6^d^	217.0^a^	168.9^c^	1.095
Valine	159.4^b^	192.4^a^	200.8^a^	154.8^b^	2.696

^a–d^ Means within a row with different superscript letters are different (*P* < 0.05).

^*a*^ DIAAS-like (%) = [(mg of digestible indispensable amino acid in 1 g of dietary protein)/mg of same indispensable AA in 1 g of reference protein)] × 100.

^*b*^ DMB = dry matter basis.

^*c*^ SEM = standard error of the mean.

**Table 7.** Digestible indispensable amino acid score (DIAAS)-like^*a*^ value for select plant-based protein by-products compared to NRC (2006) recommended allowances for adult cats at maintenance

%, DMB^*b*^	Treatment				
Indispensable amino acid	Soy flakes	Corn gluten meal	Soybean meal	Peanut flour	SEM^*c*^
Arginine	159.6^c^	84.6^d^	181.8^b^	236.8^a^	1.414
Histidine	160.1^b^	153.2^bc^	190.2^a^	141.2^c^	3.113
Isoleucine	173.9^c^	191.7^b^	211.2^a^	139.1^d^	2.304
Leucine	113.3^c^	312.1^a^	139.9^b^	107.6^c^	1.555
Lysine	290.6^b^	89.1^c^	353.6^a^	51.8^d^	5.365
Methionine	118.5^c^	294.1^a^	152.8^b^	96.6^d^	2.230
Phenylalanine	201.3^c^	305.4^a^	234.8^b^	226.0^b^	2.352
Threonine	100.4^c^	115.3^b^	137.2^a^	69.3^d^	2.172
Tryptophan	175.2^b^	92.3^d^	205.3^a^	159.8^c^	1.035
Valine	149.1^b^	179.9^a^	187.8^a^	144.4^b^	2.522

^a–d^ Means within a row with different superscript letters are different at (*P* < 0.05).

^*a*^ DIAAS-like (%) = [(mg of digestible indispensable amino acid in 1 g of dietary protein)/mg of same indispensable AA in 1 g of reference protein)] × 100.

^*b*^ DMB = dry matter basis.

^*c*^ SEM = standard error of the mean.

When the DIAAS-reference scores for the test ingredients were compared to AAFCO (2020) recommended values for dogs, the methionine content was considered moderate quality for SF (54.6%) and SBM (70.5%) but was significantly lowest (*P* < 0.05) for PF with a value of 44.5%. However, the DIAAS-like methionine value was highest (*P* < 0.05) for CGM, with a value of 135.5%. All of the test ingredients were different (*P* < 0.05) from each other for DIAAS-reference lysine scores, with PF having the lowest DIAAS-like value (25.1%). Methionine was the first-limiting amino acid for SF and SBM, while lysine was the first-limiting amino acid for PF and CGM. As mentioned previously, the decreased protein quality observed in PF could be due to the reduced availability of lysine caused by the formation of Maillard reaction products. When compared to NRC (2006) canine recommended allowances, the first-limiting amino acid for PF is methionine rather than lysine with a DIAAS-like value of 24.9%. Additionally, the number of DIAAS-reference scores below 100% increases when compared to NRC recommended allowances than AAFCO recommended values due to stricter amino acid requirements set by the NRC.

Until recently, the assessment of protein quality of ingredients used in companion animal diets has not been determined using DIAAS values. Recent studies in companion animal nutrition have used DIAAS-like values to evaluate novel protein sources (Do et al., 2020) and traditional protein sources (Oba et al., 2019) commonly used in canine and feline diets. Do et al. (2020) evaluated DIAAS-like scores of black soldier fly larvae at various ages for adult dogs and cats. Compared to AAFCO recommended values for adult dogs, the first-limiting amino acid was methionine for all larvae ages with a DIAAS-like score of 73%. Similarly, Oba et al. (2019) evaluated the DIAAS-like scores for raw chicken, retorted chicken, steamed chicken, and chicken meal. When using AAFCO recommended values for adult dogs as the reference protein, methionine was the first-limiting amino acid for a chicken meal while tryptophan was first-limiting for the other chicken ingredients (Oba et al., 2019).

Compared to AAFCO recommended values for adult cats at maintenance, all DIAAS-reference scores were over 100% for SBM indicating there is no limiting amino acid. Similarly, SF had DIAAS-reference scores over 100% for all indispensable amino acids with the exception of threonine (92.8%) which is the first-limiting amino acid. Scores below 100% were observed for arginine (81.4%) and tryptophan (97.6%) in CGM and for threonine (63.9%) in PF. Lysine was the first-limiting amino acid for CGM (47.3%) and PF (27.5%). Using NRC recommended allowances as the reference protein pattern, both SF and SBM had DIAAS-reference scores over 100% for all indispensable amino acids. While the first-limiting amino acid was lysine for PF (51.8%), the first-limiting amino acid for CGM shifted to arginine (84.6%). Similarly, Do et al. (2020) reported arginine as the first-limiting amino acid for all but one analyzed age of black soldier fly larvae for adult cats based on AAFCO recommended values. When compared to NRC recommended allowances for adult cats, the lowest DIAAS-like values for arginine and leucine were over 100%. Oba et al. (2019) determined threonine is the first-limiting amino acid in all the chicken ingredients when compared to AAFCO recommended values (91.5%) and NRC recommended allowances (98.8%) for adult cats at maintenance.

## CONCLUSIONS

Despite the variation observed in plant-based protein by-products due to processing methods and growing conditions, these ingredients have beneficial compositions and sufficient protein quality for use in canine and feline diets. The high-protein, high-fiber, and low-fat compositions of these ingredients make them easy to incorporate into pet food formulations. Their varied amino acid compositions provide an opportunity to combine them to create complementary proteins to meet the nutritional requirements of dogs and cats. However, their use must account for differences in processing parameters that may decrease protein quality due to the presence of anti-nutritional factors if under-processed or the formation of Maillard reaction products if exposed to high temperatures. Future studies are needed to better characterize the impact of these negative attributes when processed plant-based protein sources are used in canine and feline diets. Likewise, this characterization will require *in vivo* studies in dogs and cats to assess the nutritional adequacy and optimal inclusion levels of these processed ingredients in different food matrices exposed to further heat-based processing conditions.
